# Arthritis Robustus: review of a case of an “abnormal” rheumatoid

**DOI:** 10.1186/2193-1801-3-606

**Published:** 2014-10-16

**Authors:** Kajal Prasad, Deepak Rath, Bijit Kumar Kundu

**Affiliations:** Department of Medicine, PGIMER and Dr RML Hospital, New Delhi, India; Rheumatology Clinic, Department of Medicine, PGIMER and Dr RML Hospital, New Delhi, India

**Keywords:** Arthritis robustus, Atypical presentation, Rheumatoid arthritis, Rheumatoid robustus

## Abstract

**Introduction:**

Incidental discovery or diagnosis of Rheumatoid Arthritis where the patient remains blissfully unaware of his affection is a rare occurrence.

**Case description:**

We present the case of a telephone wireman in whom Rheumatoid Arthritis neither affected his activities of daily living nor caused any deformity to develop. It remained asymptomatic till its incidental discovery during his admission for treatment of myocardial infarction.

**Discussion and Evaluation:**

This presentation of Rheumatoid Arthritis is termed ‘Arthritis Robustus’ and goes against the very tenets of the picture of Rheumatoid Arthritis we have in our minds. The name given to this entity stems from the fact that these patients are mostly physical labourers i.e. ‘Robust’.

**Conclusion:**

Rheumatoid Arthritis can very rarely be asymptomatic. The rarity of the entity can be inferred from the paucity of published literature.

## Background

Pain and stiffness are the classical presenting symptoms of Rheumatoid Arthritis (RA), and along with small joint affection, and relevant investigations thus prompted make the diagnosis obvious in most cases. Rarely however, a patient of RA may be entirely symptom free and is only detected incidentally when under evaluation for another symptom or disease. We present such a case where an elderly gentleman was detected to be having such a variant of RA while being treated for symptomatic coronary artery disease (CAD).

### Case description

**Figure 1:**
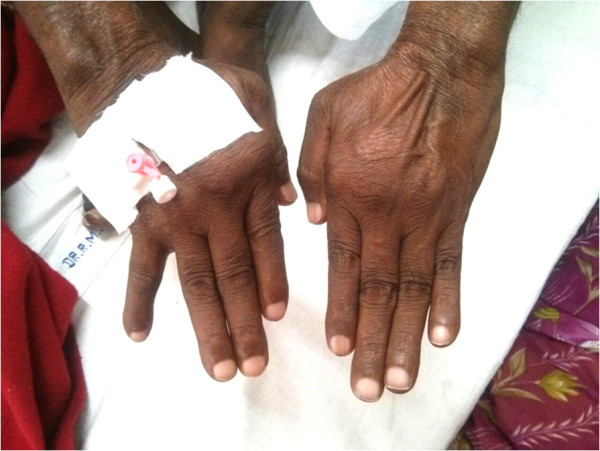
**Swollen metacarpopahalangeal joints.**

**Figure 2:**
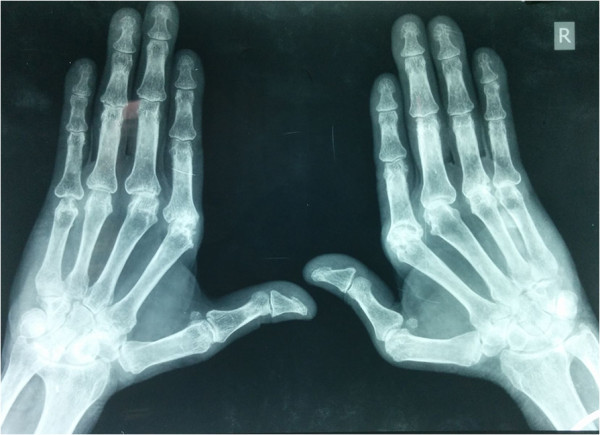
**Radiograph of both hands.** Note the prominent erosion in the left 2nd MCP.

Mr G, a 58 year male, telephone wireman by profession, with an active lifestyle was admitted with the diagnosis of Non ST Elevated Myocardial Infarction (NSTEMI) with the only risk factor being smoking (Ten pack years). We noticed swollen but non tender metacarpophalangeal (MCP) joints. Musculoskeletal examination revealed bilaterally symmetrical synovial swelling and deformity of the MCPs of the hands which were reducible (Figure 1). He denied ever having any pain, stiffness, or difficulty in carrying out activities of daily living (ADL); and hence there was no reason to present to a physician. We investigated him in light of his bilateral symmetrical synovitis in addition to the investigations related to the primary diagnosis of CAD. His blood counts were normal, Erthrocyte Sedimentation Rate (ESR) was high of 80 mm/1st hour (Westergren’s method), and liver and kidney functions were normal. Lipid profile values were within normal limits. C - reactive protein (CRP) was raised, Rheumatoid Factor (RF) and anti cyclic citrullinated peptide antibodies (Anti CCP) were positive in high titres, >120 IU/mL and >200 U/mL respectively. His X-ray hands are shown below (Figure 2). His diagnosis was kept as CAD (NSTEMI) with Arthritis Robustus, a variant of RA, with risk factors of CAD being smoking and RA. In addition to the treatment of CAD, treatment of RA was also initiated with methotrexate and hydroxychloroquin.

## Discussion

Classically RA presents as gradual onset, slowly progressive polyarthritis affecting the small joints of the upper limb more than the lower limb in a symmetrical pattern. The acute onset pattern is also well known. Other variations in onset and pattern are, the Palindromic onset, the insidious onset in the elderly, and the rarer Rheumatoid Nodulosis, and the Arthritis Robustus (Sweeney et al. 2013).

Arthritis Robustus, also called Rheumatoid Robustus was first described by de Haas WH et al. in 1973 who described this variant in patients who were robust and hence the name (de Haas WH et al. 1973). It mostly affects men who are active, and are involved in professions entailing physical labour. It is thought to be an unusual reaction of the patient to the disease. Pain and stiffness are absent or at most negligible, though deformities may occur. Neither disability is present nor ADL are affected and patients move through life blissfully unaware of their disease till its incidental diagnosis. The most significant finding on examination is synovial proliferation. Subcutaneous nodules may be present and can be large. Radiological findings include prominent erosions, with new bone formation at joint margins near the erosions and subchondral cysts, presumed to be due to excessive pressure caused by muscular effort on the synovial fluid within a thick joint capsule. However, periarticular osteopenia is rare in comparison to classical RA.

Our patient was a telephone wireman whose job profile entailed continuous use of the small joints of his hand. He kept working at his job, his symptoms never coming to his notice. The only risk factor for his CAD was his being a smoker. Interestingly, smoking is not only a risk factor for CAD, but also for development of seropositive RA. This risk is dependent on the amount of smoking in pack years and contributes to 35% of the Anti–citrullinated protein antibody (ACPA) positive cases, as well as to more than 55% of ACPA positive cases in individuals with two copies of the shared epitope Human Leucocyte Antigen (HLA) DRβ1 (Källberg et al. 2011). Interestingly, the risk does not increase further with exposure higher than 20 pack years (Di Giuseppe et al. 2014). Besides, RA is also a risk factor for CAD (Warrington et al. 2005), probably due to the expansion of the CD4 + CD28^null^ T-cells, and treatment of RA decreases the incidence of CAD. Thus our patient had two risk factors of CAD, namely smoking and RA.

The rarity of this entity can be gauged from the fact that a search in PUBMED database reveals only two publications in addition to the original description in the last four decades. This entity goes against the mental picture we have of RA viz, painful joints with restriction of movement affecting ADL and making a person disabled; and therein lays its novelty. Our case also underlines the interrelation between RA, smoking, and CAD, with smoking being a risk factor of both, and RA being a risk factor for CAD.

## Conclusions

Rheumatoid arthritis can very rarely be asymptomatic; hence may be undetected, except incidentally.

## Consent

Written informed consent in the patient’s own mother tongue was obtained from the patient for the publication of this report and any accompanying images.

No experimental research is reported in this case study.

## Authors’ information

**KP** (MBBS) is doing her residency in the Department of Medicine, PGIMER & Dr Ram Manohar Lohia Hospital, New Delhi, India. She takes an active interest in rheumatologic-patient care.

**DR**: (MD) is Senior Resident at Department of Medicine, PGIMER & Dr Ram Manohar Lohia Hospital, New Delhi, India. He plans to peruse super-specialization in Rheumatology and Clinical Immunology.

**BKK** (MD) works in Department of Medicine, PGIMER, Dr RML Hospital, New Delhi, India. He conducts the Rheumatology Clinic. He practices in a government institution with the aim of making rheumatology services accessible to the poorer sections of the society who cannot afford them in the private sector. He mainly focuses on conducting talks for general practitioners, the point of first contact for the patient, to increase the awareness and penetration of rheumatology.
